# The potential link between acromegaly and risk of acute ischemic stroke in patients with pituitary adenoma: a new perspective

**DOI:** 10.1007/s13760-023-02354-3

**Published:** 2023-08-16

**Authors:** Saud A. Alnaaim, Hayder M. Al-kuraishy, Mohammad Mitran Zailaie, Athanasios Alexiou, Marios Papadakis, Hebatallah M. Saad, Gaber El-Saber Batiha

**Affiliations:** 1 Clinical Neurosciences Department, College of Medicine, King Faisal University, Hofuf, Saudi Arabia; 2 Department of Pharmacology, Toxicology and Medicine, Medical Faculty, College of Medicine, Al-Mustansiriyah University, PO Box 14132, Baghdad, Iraq; 3 Prince Mohammed Bin Nasser Hospital, Jazan, Saudi Arabia; 4 Department of Science and Engineering, Novel Global Community Educational Foundation, Hebersham, NSW 2770 Australia; 5 AFNP Med, 1030 Vienna, Austria; 6 Department of Surgery II, University Hospital Witten-Herdecke, University of Witten-Herdecke, Heusnerstrasse 40, 42283 Wuppertal, Germany; 7 Department of Pathology, Faculty of Veterinary Medicine, Matrouh University, Marsa Matruh, 51744 Egypt; 8 Department of Pharmacology and Therapeutics, Faculty of Veterinary Medicine, Damanhour University, Damanhour, 22511 Egypt

**Keywords:** Acromegaly, Acute ischemic stroke, Growth hormone, Insulin-like growth factor 1

## Abstract

Acromegaly is an endocrine disorder due to the excess production of growth hormone (GH) from the anterior pituitary gland after closed epiphyseal growth plates. Acromegaly is mainly caused by benign GH-secreting pituitary adenoma. Acute ischemic stroke (AIS) is one of the most common cardiovascular complications. It ranks second after ischemic heart disease (IHD) as a cause of disability and death in high-income countries globally. Thus, this review aimed to elucidate the possible link between acromegaly and the development of AIS. The local effects of acromegaly in the development of AIS are related to the development of pituitary adenoma and associated surgical and radiotherapies. Pituitary adenoma triggers the development of AIS through different mechanisms, particularly aneurysmal formation, associated thrombosis, and alteration of cerebral microcirculation. Cardiovascular complications and mortality were higher in patients with pituitary adenoma. The systemic effect of acromegaly-induced cardio–metabolic disorders may increase the risk for the development of AIS. Additionally, acromegaly contributes to the development of endothelial dysfunction (ED), inflammatory and oxidative stress, and induction of thrombosis that increases the risk for the development of AIS. Moreover, activated signaling pathways, including activator of transcription 3 (STAT3), nuclear factor kappa B (NF-κB), nod-like receptor pyrin 3 (NLRP3) inflammasome, and mitogen-activated protein kinase (MAPK) in acromegaly may induce systemic inflammation with the development of cardiovascular complications mainly AIS. Taken together, acromegaly triggers the development of AIS through local and systemic effects by inducing the formation of a cerebral vessel aneurysm, the release of pro-inflammatory cytokines, the development of oxidative stress, ED, and thrombosis correspondingly.

## Introduction

**Fig. 1:**
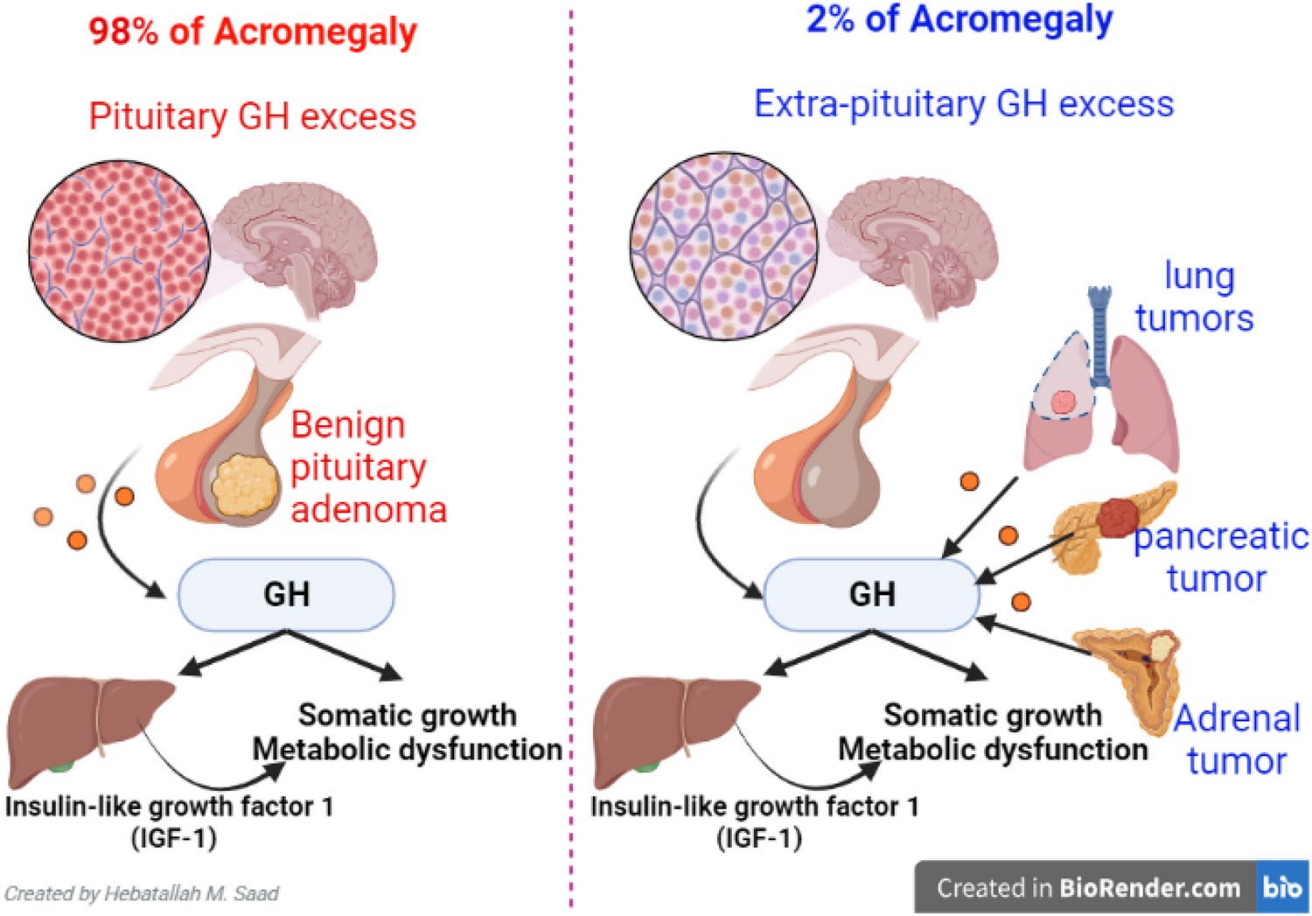
The pathophysiology of acromegaly

Acromegaly is an endocrine disorder due to excess growth hormone (GH) production from the anterior pituitary gland after epiphyseal growth plates have closed [1]. The pathophysiology of acromegaly is summarized in Fig. 1. Acromegaly is mainly caused in about 98% by benign pituitary adenoma [1]. 2% of acromegaly is caused by excessive extra-pituitary production of GH, as in adrenal, pancreatic, and lung tumors [2]. It affects 3 per 50,000 with equal incidence in both sexes [3, 4].

Acromegaly was first REPORTED by the French Neurologist Pierre Marie in 1886 to describe the clinical features of two patients observed at the Pitié-Salpetriere Hospital in Paris. However, Pierre Marie was not the first physician to give a full record of the clinical picture of acromegaly; others had preceded him, like the Dutch physician Johannes Wier [5]. After Marie, pituitary enlargement was noted in almost all patients with acromegaly. Subsequently, it was discovered that pituitary hyper-function caused by a pituitary tumor was the main cause of acromegaly. The cause of acromegaly could be further determined after discovering GH and insulin-like growth factor I (IGF-I) and demonstrating an association with GH hypersecretion and elevated circulating IGF-I [6].

The majority of pituitary tumors are sporadic, and more than half have no identified genetic cause. However, in somatotroph cells, genetic abnormalities in proteins involved in the cyclic AMP pathway (such as GNAS, AIP, GPR101, and PRKAR1A) contribute to increasing AMP signaling, which leads to increased GH expression and cell proliferation. Familial pituitary tumors are rare and constitute approximately 5% of all pituitary adenomas. Multiple endocrine neoplasia type 1, multiple endocrine neoplasia type 4, familial isolated pituitary adenoma, and Carney complex, as well as the sporadic germline mosaic disorder and McCune-Albright disease, are among familial syndromes that predispose to pituitary hyperplasia and neoplasia, causing acromegaly or gigantism [7]. A rare genetic syndrome, X-linked acrogigantism, has also been recently implicated in early-onset childhood gigantism [7, 8].

Expansion of pituitary adenoma compresses the optic nerve and other brain tissues leading to visual disturbances, hormonal disorders, headache, impotence, and enlargement of the hand and feet. The main symptoms and signs of acromegaly are enlargement of feet, hands, jaws, forehead, and nose. Other features of acromegaly are thick skin, joint pain, and deepening of voice [9].

In general, signs and symptoms of acromegaly develop insidiously and are unnoticed for many years. However, more than 70% of patients present with pituitary macroadenoma at the time of diagnosis, and more than 90% present with complications due to the diagnostic delay [10]. Headache, coarse facial features, acral growth, and sweating/oily skin are the most frequent presenting complaints. The most common comorbidities are diabetes mellitus, hypertension, sleep apnea, hypogonadism, and cardiomyopathy [11, 12].

Mutations and genetic deregulation within the pituitary affect the cellular chemical signals and subsequent division and proliferation of pituitary cells [11, 13]. Moreover, aryl hydrocarbon receptor (AHR) polymorphism is associated with the development and progression of acromegaly. A case–control study that involved 70 patients with acromegaly and 157 sex and aged-matched controls showed that the *rs2066853* polymorphism was detected in 18 acromegalic patients and 9 healthy controls (*P* < 0.001) [13]. Therefore, AHR gene *rs2066853* polymorphism is more common in acromegalic patients than in healthy individuals and is linked with increased disease severity. As well, functional alterations of genetic derangements may associate with early-onset acromegaly. Deletion in *CDKN1B* 5′-UTR region was recognized in an acromegalic patient that developed a growth hormone-secreting pituitary adenoma during childhood [14].

Diagnosis of acromegaly is performed by measuring GH and IGF-1; besides, imaging studies like brain computed tomography (CT) scan and magnetic resonance tomography imaging (MRI) were used to show the size and extension of pituitary adenomas [15]. The biochemical diagnosis of acromegaly is traditionally based on over-secreted GH and IGF-1 levels. However, in normal healthy individuals, the levels of circulating GH secreted from the pituitary fluctuate greatly throughout the day owing to the pulsatile nature of GH production [15]. Maximum secretion of GH occurs at night in accordance with sleep stages. The value of GH usually ranges between 0.1 and 0.2 µg/L and 5–30 µg/L during the secretory bursts, and these values overlap with the values observed in acromegaly patients. A random GH value < 0.04 µg/L with a normal level of IGF-1 excludes the diagnosis. Therefore, determining the GH level is of minimal diagnostic utility in acromegaly. Instead, GH level < 0.4 ng/mL in an oral glucose tolerance test is the gold standard for diagnosis. In normal individuals, the GH nadir value during an oral glucose tolerance test (OGTT) is undetectable as secretion is suppressed, but the value is very high in acromegaly patients owing to the lack of suppression [15].

Pituitary surgery is the first-line treatment option (trans sphenoidal surgery is the primary therapy in most patients), followed by medical treatment, including somatostatin analogs, dopamine agonists, and GH receptor antagonists [16]. In addition, radiation therapy is recommended in the setting of residual tumor mass following surgery, and if medical therapy is unavailable, unsuccessful, or not tolerated [15, 16].

High levels of GH and IGF-1 are associated with mortality in patients with acromegaly [17]. In addition, acromegaly patients are at high risk for developing congestive heart failure and atrial fibrillation in a time-dependent manner [18]. A recent retrospective study confirmed the association between acromegaly and the incidence of cardiovascular complications like congestive heart failure and atrial fibrillation [18]. Data from the German Acromegaly Registry Center showed that the incidence of cardiovascular complications in patients with acromegaly did not differ significantly from that of the general population. Acromegaly is also associated with established risk factors for cardiovascular diseases, such as hypertension, diabetes, and dyslipidemia [19]. A meta-analysis of 16 studies in 2008 showed that acromegaly is associated with an overall 72% increase in mortality compared with the general population [20].

AIS is regarded as one of the most common cardiovascular complications of systemic hypertension and thrombotic disorders [21]. Hypertension and thrombotic disorders are often recognized as complications of acromegaly [12]. Thus, this review aimed to elucidate the possible link between acromegaly and the development of AIS.

## Acute ischemic stroke overview

**Fig. 2:**
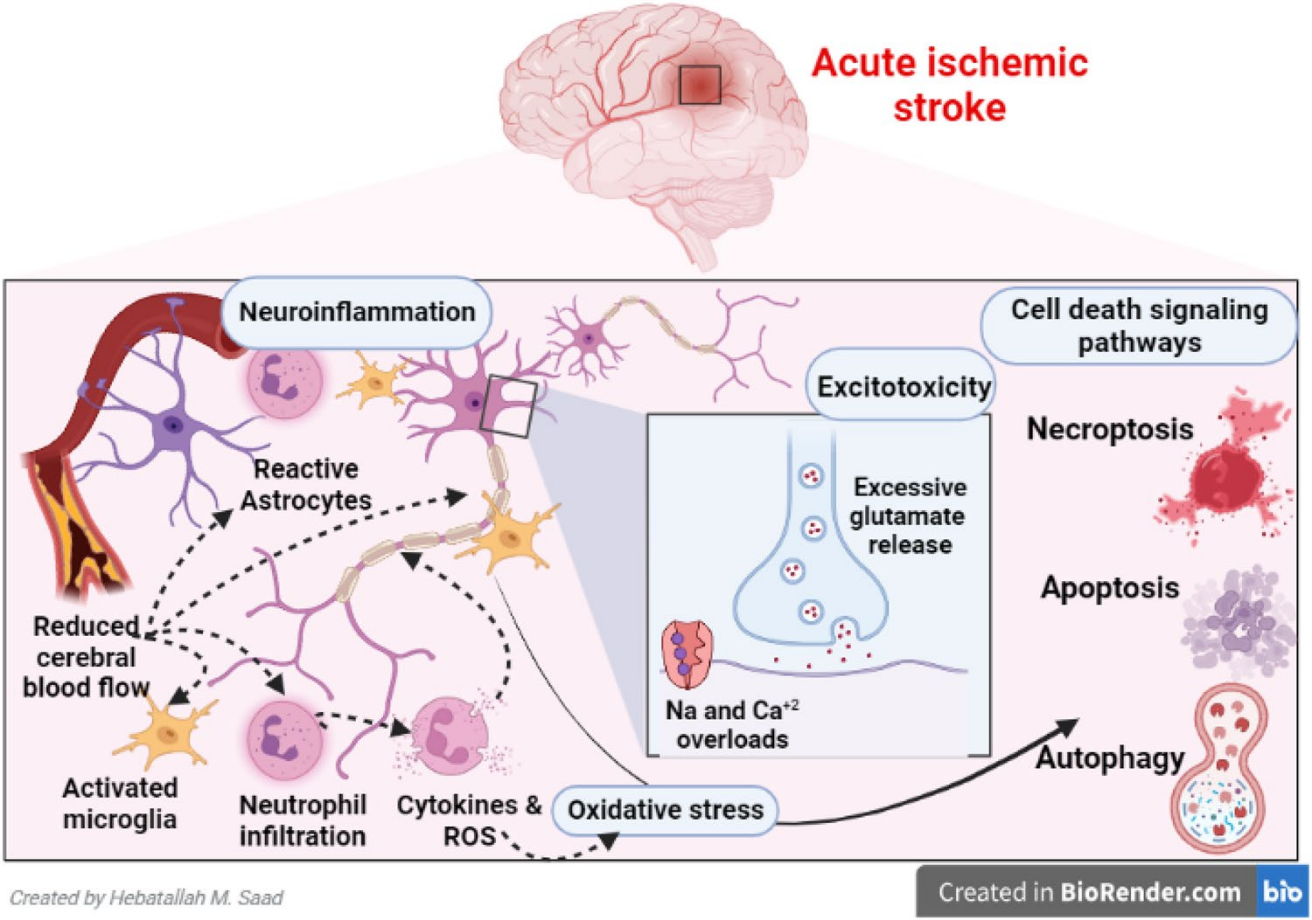
The pathophysiology of acute ischemic stroke

AIS ranks second, following ischemic heart disease (IHD) as a cause of disability and death in high-income countries globally [22]. The incidence of AIS varies among countries; it increases exponentially with age. About 80% of stroke is caused by AIS, whereas 20% of it is caused by intra-cerebral hemorrhage [22]. The poor clinical outcomes in AIS patients are correlated with cardiometabolic risk factors like infarct size, hypertension, IHD, and diabetes mellitus [23]. AIS is principally caused by atherosclerosis, rupture of atherosclerotic plaque, and associated thrombosis of cerebral vasculatures, leading to brain ischemia, infarction, and propagation of peri-infarct inflammation with induction development of neuroinflammation [24]. AIS-induced neuroinflammation triggers neuronal injury and axonal degeneration (Fig. 2) [25].

Neuronal injury in the infarcted core provokes the release of pro-inflammatory cytokines with the propagation of oxidative stress, vasculitis, and derangement of the blood–brain barrier (BBB) [26]. Markedly, BBB is an important part of neurovascular units containing tight junctions, different ions, and proteins that promote brain function and physiology [27]. Disruption of BBB is a principal pathological hallmark in AIS that is dysregulated by inflammatory modulators, matrix metalloproteinases (MMPs), and oxidative pathways [28]. Interruption of BBB worsens AIS injury with the propensity for transformation to hemorrhagic stroke [29]. In AIS, activated macrophages and microglia lead to the disturbance of neurotransmitters and the development of neuroinflammation. AIS-induced neuroinflammation is propagated via the release of interleukins (IL-1β), IL-6, IL-8, and tumor necrosis factors (TNF-α) as well as activation of inflammatory signaling pathways like nuclear factor kappa B (NF-κB) [29]. These changes promote apoptosis and neuroinflammation. Remarkably, neuronal injury and associated neuroinflammation disrupt the neuronal cyclic adenosine monophosphate (cAMP) system which is important for the expression of phosphodiesterase enzyme type 1 (PDE1) and brain-derived neurotrophic factor (BDNF) [30]. Furthermore, insulin resistance, diabetes mellitus, and hypertension secondary to acromegaly predispose to developing AIS. Cardiometabolic disorders and dysregulation of adipocytokines also augment AIS risk.

Taken together, AIS, in addition to causing brain neuronal injury, may exacerbate the development of neuroinflammation and excitotoxicity with disruption of neuronal ionic pumps.

## Acromegaly and risk of AIS

### Local effects of pituitary adenoma

The local effects of acromegaly in the development of AIS are related to pituitary adenoma development, which affects brain integrity of brain circulation [31]. A population-based study from Korea involving 31,898 patients with pituitary adenoma illustrated that cardiovascular complications and mortality were higher among patients with pituitary adenoma [31]. Similarly, the incidence of AIS in patients with pituitary adenoma was augmented [31]. Thus, patients with pituitary adenoma must take AIS preventive measures to decrease mortality risk. However, a retrospective review comprising 544 patients, 347 patients with prolactinoma, and 197 patients with non-functional pituitary adenoma (NFPA) showed that prolactinoma and NFPA were not increasing the risk of AIS [32]. Of interest, different studies implicate prolactin in the pathogenesis of AIS [33, 34]. Prolactin has been recognized as a potent platelet aggregation co-activator and postulated as an additional risk factor for arterial and venous thrombosis. Administration of antipsychotics appears to be related to an increased risk of venous thromboembolism and cerebrovascular by increasing the release of prolactin [34]. In addition, increased prolactin concentration is significantly linked with augmentation of platelet P-selectin in patients with transient ischemic attack [33]. These consequences proposed that patients with high prolactin levels after AIS may have a worse prognosis.

Even though AIS and hemorrhagic stroke may result from the development of a cerebral aneurysm [35], pituitary adenoma is not associated with the development of an aneurysm in brain circulation [36]. However, Habibi et al. [37], in a case study, observed that pituitary adenoma may coexist with an aneurysm in the posterior brain circulation causing hemorrhagic stroke and AIS during trans sphenoidal surgery. Thus, neuroimaging of brain circulation before the surgical intervention of pituitary adenoma would be helpful to rule out vascular anomalies in the brain vasculatures [37]. Besides, a neuroimaging study in a 52-year-old patient with pituitary adenoma detected an aneurysm of the internal carotid artery [38]. It has been shown that pituitary adenoma is linked with developing a cerebral aneurysm. Notably, Wakai et al. [39] analyzed 95 patients with pituitary adenoma and revealed that the incidence of the cerebral aneurysm was 7.45% which was higher than other brain tumors. Similarly, an angiographic study that included 150 verified pituitary adenomas showed that pituitary adenoma secreting GH was linked with developing an aneurysm by about 13.8% [40].

The association between pituitary adenoma secreting GH and the development of cerebral aneurysms is related to the progression of arteriosclerosis and cerebral degenerative changes due to prolonged exposure to GH [41]. Besides, local inflammatory/circulatory mediators released from pituitary adenoma may induce structural changes through modulation of arterial collagen metabolism with the promotion formation of the aneurysm [38]. A previous retrospective study conducted by Pant et al. [36] illustrated that pituitary adenoma is associated with 5.4% of intracranial aneurysms, 97% of them present in the anterior circulation, and 12% located in different parts of cerebral circulation. Different studies confirmed that the aneurysm of cerebral circulation may induce the development of AIS by interrupting cerebral blood flow [42, 43]. Of interest, the cerebral aneurysm may act as a nidus for thrombosis and the development of ipsilateral AIS due to hemodynamic and endothelial alterations [42, 44]. A systematic review revealed a potential link between acromegaly and endothelial dysfunction (ED), which increases cardiovascular risk factors and vascular anomalies [44]. However, a case–control study that included 2116 patients with AIS showed no association between cerebral aneurysms and AIS [42].

Nonetheless, many clinical studies confirmed that platelet and clotting factor over-activations are associated with thrombosis within cerebral aneurysms [45, 46]. Furthermore, expansion of pituitary adenoma is associated with hypoperfusion and ischemic changes in the brain's temporal lobe by direct compression of regional blood flow [47]. A single photon emission computed tomography (SPECT) study demonstrated that temporal lobe blood flow was reduced in patients with pituitary adenoma compared to healthy controls [47].

These findings highlighted that pituitary adenoma may trigger the development of AIS through different mechanisms, particularly aneurysmal formation, associated thrombosis, and alteration of cerebral microcirculation.

Management of pituitary adenoma by surgical or radiotherapy techniques may increase the risk of AIS development [48]. Eseonus et al. [49] revealed that trans sphenoidal surgery for pituitary adenoma may cause cerebral vasospasm and progression of AIS. A retrospective case report confirmed that post-operative hemorrhage and cerebrospinal fluid (CSF) leakage could be the possible causes for the development of cerebral vasospasm and propagation of AIS [49]. In addition, pituitary apoplexy following AIS may induce cerebral vasospasm and ischemia [50].

Remarkably, radiotherapy for pituitary adenoma may accelerate the progression of AIS due to cerebral vasculopathy [48]. A cohort study included 462 patients with pituitary adenoma, 236 of them treated with radiotherapy, and disclosed that radiotherapy used in the management of pituitary adenoma was correlated with the risk of AIS more significantly compared to the general population [51]. A systematic review comprising 11 studies including 4394 irradiated patients with pituitary adenoma illustrated that the risk of AIS was 6.7% [52]. Long-term follow-up of irradiated patients with pituitary adenoma revealed that the risk of AIS was elevated threefold [52]. However, corrections of many confounders confirmed the insignificant difference between radiated and non-radiated patients with pituitary adenoma for risk of AIS [52]. The relative risk for developing AIS following irradiation of pituitary adenoma is 4.11% [53]. Sub-analysis performed by Van et al. [54] observed that the risk for AIS progression following pituitary adenoma irradiation was higher threefold, mainly in men patients. The underlying causes for developing AIS following radiotherapy of pituitary adenoma could be related to the vascular injury of surrounding vessels [55]. Radiation-induced vascular injury is mediated by the induction expression of pro-inflammatory cytokines and hypoxia-inducible genes with the development of endothelial loss [55]. These pathological changes trigger thromboembolic events and the development of AIS [54]. Interestingly, the type of radiotherapy and follow-up time may affect the incidence and prevalence of AIS after irradiation of pituitary adenoma [52].

These findings suggest that local effects of pituitary adenoma and associated surgical and radiotherapies may increase the risk for the development of AIS.

### Systemic effects of acromegaly

High circulating GH levels in acromegaly may predispose to the development of AIS. Following the diagnosis of acromegaly, irrespective of lifestyle and treatment types, are regarded as predictors for the progression of cardiovascular events. Systemic cardio metabolic disorders like hypertension, diabetes mellitus, and dyslipidemia are higher in acromegaly patients than in general. These predisposing factors are concerned with the induction of AIS. Notably, higher concentrations of GH and IGF-1 in acromegaly increase the risk of cardiovascular complications. Excess GH level is associated with the development of IHD, glucose intolerance and diabetes mellitus, whereas a higher concentration of IGF-1 is linked with the development of cardiomyopathy and AIS. Besides, increasing GH levels in acromegaly augments intramuscular deposition of adipose tissue, which impairs glucose sensitivity and the propagation of cardio-metabolic disorders [56].

Notoriously, patients with active acromegaly had a higher rate of classical and non-classical cardiovascular risk factors, which may explain the association between acromegaly and cardiovascular events, including AIS [57]. A case–control study involved 50 patients with active acromegaly, 12 patients with controlled acromegaly, and 36 healthy control subjects. This study demonstrated that in comparison with controlled acromegaly and healthy subjects, patients with active acromegaly had higher values of cholesterol, triglyceride, low-density lipoprotein (LDL), very low-density lipoprotein (VLDL), fibrinogen, protein S, and fasting blood glucose [57]. Remarkably, strict control of acromegaly may not prevent the progression of subclinical atherosclerosis [58]. Thus, excess GH/IGF-1 is not the main driver of subclinical atherosclerosis in acromegaly patients. Therefore, appropriate therapy and control of acromegaly may not reverse the abnormality of vasculature structure [58]. A case–control study observed that the prevalence of carotid atherosclerotic plaques and intima-media thickness was similar in both controlled and active acromegaly patients (30% vs. 22%, *P* = 0.53) [58]. Therefore, other factors like lifestyle, diet, and physical activity than GH/IGF-1 levels may play a role in the development of cerebrovascular diseases, mainly AIS, in patients with acromegaly. These verdicts indicated that acromegaly-induced cardio metabolic disorders may increase the risk for the development of AIS, even in controlled patients.

## Acromegaly and thrombosis

Hypersecretion of GH and IGF-1 in acromegaly increases cardiovascular complications, morbidity, and mortality due to coagulation activation and fibrinolytic system impairments [59]. Of note, fibrinolysis inhibitors and plasma tissue factor pathway inhibitors are dysregulated in patients with active acromegaly [59]. A case–control study involved 22 patients with active acromegaly and 22 matched healthy controls showed that fibrinogen, antithrombin III, tissue plasminogen inhibitor 1, and plasminogen activator inhibitor 1 (PAI-1) were increased, while protein S and plasma tissue factor pathway inhibitor were reduced in active acromegaly patients compared with controls [59]. Thus, active acromegaly is associated with a hypofibrinolytic and hypercoagulable state which augments the risk of AIS development. Chuang et al. [60] reported that fibrinogen was regarded as an independent predictor for the development of AIS. Likewise, augmentation of fibrinogen level and reduction of antithrombin III are correlated with the risk of AIS development [61]. A prospective study of AIS patients illustrated that fibrinogen and thrombin-antithrombin III levels were increased in AIS patients compared with non-stroke patients [61]. However, high sensitive C reactive protein (CRP) and D-dimer were increased in a small proportion of AIS patients with large vessel infarction [61]. These manifestations pointed out those focal neurological deficits in AIS patients are positively correlated with fibrinogen levels and negatively correlated with antithrombin III. Mendoza et al. [62] reported a case of intracardiac thrombus with the development of cardiometabolic AIS in patients with pituitary adenoma. Congestive heart failure and cardiomyopathy induced by high circulating GH in pituitary adenoma predispose to the development of intracardiac thrombus [62]. In addition, acromegaly patients may present with massive pulmonary embolism due to the hypercoagulability state [63]. Hypercoagulability state in acromegaly is caused by platelet activation, hyperfibrinogenemia, and reduction of proteins C and S [63].

Indeed, plasma thrombin-activatable fibrinolysis inhibitor (TAFI) antigen and homocysteine serum levels are increased in patients with active acromegaly that augments cardiometabolic events, including AIS [64]. Thus, acromegaly has an increased tendency to thrombosis and coagulation disorders with the development of cerebrovascular complications [65]. A case–control study comprising 39 patients with active acromegaly compared to 35 healthy controls illustrated that fibrinogen level was increased, whereas proteins C and S were reduced in patients with active acromegaly compared to the controls [65]. Also, a positive correlation exists between GH/IGF-1 levels and fibrinogen levels in active acromegaly [44]. Moreover, excess morbidity and mortality observed in patients with acromegaly are not only due to the impairment of the fibrinolytic system but also due to many other factors. In particular, diabetes and cardiovascular diseases are important determinants of mortality and may also impact cerebrovascular diseases [66]. An observational, matched cohort study conducted by Esposito et al. [66] involving patients with acromegaly between 1987 and 2020 showed that the development of diabetes in patients with acromegaly was linked with a higher risk for cardiovascular morbidity and mortality.

Moreover, acromegaly is associated with the development of oxidative stress and ED which are the underlying mechanism for the progression of atherosclerosis and cardiovascular events [44, 67]. Different studies illustrated that functional and morphological vascular changes are mainly related to high GH/IGF-1 levels independent of the conventional risk factors. GH excess leads to ED with direct and detrimental effects on the vascular wall [68, 69]. A prospective study involving 15 patients with active acromegaly compared to 15 matched healthy controls revealed that biomarkers of ED and oxidative stress and reduced endothelial nitric oxide (NO) are increased and correlated with GH/IGF levels [67]. Acromegaly is associated with increased levels of oxidative stress coupled with diminished antioxidant capacity and endothelial dysfunction indicated by decreased NO levels [67]. Besides, ED and persistent inflammation are developed in patients with active acromegaly leading to oxidative stress [68]. A cross-sectional study comprising 71 patients with active acromegaly and 41 matched healthy controls showed that vascular inflammation and ED mediators were increased compared to the controls. However, despite active therapy, these biomarkers were not mitigated. Thus, persistent inflammation and ED in active acromegaly contribute to cardiovascular complications, including AIS [70]. Particularly, oxidative stress, inflammation, and ED are associated with the increasing development of thrombosis [71]. There is positive loop interaction between ED, inflammatory mediators, and oxidative stress, causing thrombosis and cardiovascular complications [71]. Different studies confirmed the association between ED, inflammatory and oxidative stress with the development of AIS [72, 73].

**Fig. 3:**
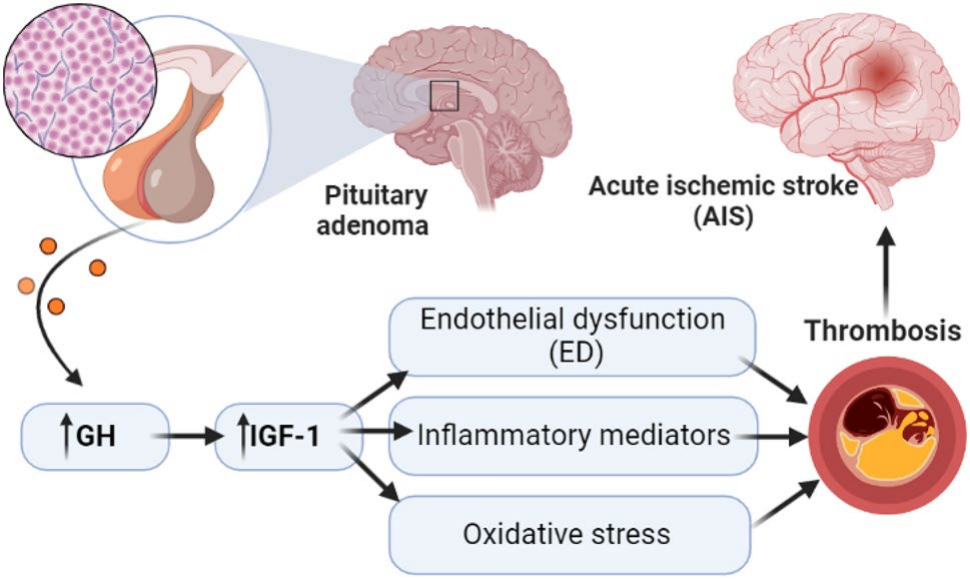
Acromegaly and thrombosis: Hypersecretion of growth hormone (GH) and insulin-like growth factor I (IGF-I) in acromegaly is associated with the development of inflammatory, oxidative stress, and endothelial dysfunction (ED) together with induction of thrombosis may increase the risk for the development of acute ischemic stroke (AIS)

These findings suggest that ED, inflammatory and oxidative stress, and induction of thrombosis may increase the risk for the development of AIS (Fig. 3).

### Acromegaly and inflammatory pathways

It has been shown that acromegaly is associated with the induction of various inflammatory signaling pathways [74]. For example, GH excess triggers the activation of signal transducer and activator of transcription 3 (STAT3) and Janus kinase (JAK) in the development of hepatocellular carcinoma [74]. Inhibition of the STAT3 pathway prevents the detrimental effects of GH on insulin sensitivity in mice [75]. Besides, the STAT3 pathway is augmented in AIS, causing apoptosis and neuronal injury. Inhibition of this pathway by L-carnosine attenuates inflammatory disorders in experimental rats with AIS [75].

Moreover, AIS-induced neuroinflammation is propagated by releasing IL-1β, IL-6, IL-8, and TNF-α and activating inflammatory signaling pathways like NF-κB [76]. These changes promote apoptosis and neuroinflammation. NF-κB enhances cerebral vasoconstriction and thrombosis with further deterioration of AIS. In acromegaly, the pro-inflammatory phenotype with higher NF-κB activation, mainly in uncontrolled patients, is predominant and linked with cardiovascular complications [77, 78]. Treatment of acromegaly partially reverses pro-inflammatory complications and systemic inflammation [77]. NF-κB is exaggerated in active acromegaly by the effect of IGF-1; thus, inhibition of IGF-1 by octreotide inhibits NF-κB expression with attenuation of systemic inflammation [79]. A prospective study demonstrated that pro-inflammatory cytokines and adhesion molecules like E-selectin and vascular cell adhesion molecules 1 (VCAM-1) were increased in patients with active acromegaly compared to controlled acromegaly [77]. Therefore, un-reversed and persistent systemic inflammation in patients with active acromegaly may increase the risk of AIS and other cardiovascular complications. A prospective study involving 31 patients with active acromegaly compared to 25 healthy controls showed that TNF-α, IL-6, and activin-A were elevated in patients with active acromegaly compared to controls [80]. Targeting inflammatory signaling in acromegaly can attenuate but not prevent the development of cardiovascular derangements.

Furthermore, nod-like receptor pyrin 3 (NLRP3) inflammasome is an integral inflammatory signaling pathway involved in the pathogenesis of AIS and related neuroinflammation. Inhibition of NLRP3 inflammasome by ephedrine reduces infarct size and release of pro-inflammatory cytokines in rats. Besides, in vitro and in vivo findings revealed that NF-κB and mitogen-activated protein kinase (MAPK) is engaged with the activation of NLRP3 inflammasome during the pathogenesis of AIS [81]. Interestingly, MAPK and NF-κB are pivotal in expressing NLRP3 inflammasome in cortical neurons under ischemic/hypoxic conditions [81]. It has been shown that NF-kB growth-promoting effects appear to be facilitated by GH and IGF-1. These stimulatory effects of GH and IGF-1 on NF-kB activity are supported by observational evidence in humans; a number of individuals carrying mutations that alter NF-kB function exhibit growth failure and GH insensitivity [82]. A previous experimental study demonstrated that GH increases lung NF-κB activation and lung microvascular injury induced by lipopolysaccharide in rats [83].

On the other side, GH regulates the expression of different inflammatory signaling pathways through the activation of GH receptors. For example, a recent study by Wolters et al. [84] observed that GH deficiency or excess triggers an inflammatory process through activation expression of macrophage NLRP3 inflammasome [84]. Circulating IGF-1 inhibits GH secretion via direct negative feedback on the pituitary and also indirectly via stimulating hypothalamic somatostatin secretion [85].

Interestingly, IGF-1 excess also leads to overexpression of cell adhesion molecules, which possess several pro-inflammatory properties, a feature of microvascular inflammation leading to ED. IGF-1 excess is assumed to be a complementary pathogenic factor for ED, implying that initial endothelial damage by oxidized LDL or shear stress is mandatory for initiating IGF-1 attenuated endothelial damage via its mitogenic properties [86]. Taken together, microvascular inflammation and early atherosclerotic changes, especially ED, are more prevalent in acromegaly compared to healthy controls and are only partly reversible with disease control. Additional factors present that may contribute to ED in acromegaly patients are cardiovascular comorbidities as hypertension and metabolic disturbances, concomitant hormonal disturbances such as hypogonadism, increased levels of pro-inflammatory cytokines and expression of adhesion molecules, disturbed endothelial repair mechanisms, and vascular alterations caused by the proliferation of vascular smooth muscle cells [84].

Therefore, the depletion of GH receptors attenuates and protects against the development of age-mediated immune senescence and NLRP3 inflammasome stimulation [87]. Deletion of GH-R prevented macrophage-driven age-related inflammasome activation in response to NLRP3 ligands. Also, it increased the preservation of naive T cells, even in advanced age and with higher interferon Gamma (IFNγ) secretion from effector cells. The mechanism of inflammasome inhibition is linked to the autocrine somatotropic axis as ablation of IGF1R in macrophages lowered the NLRP3 inflammasome activation [87]. Similarly, inhibition of macrophage IGF-1 receptors abolishes NLRP3 inflammasome activation and release of pro-inflammatory cytokines [86]. Thus, targeting the somatotropic axis in macrophages could be a pathway for preventing GH/IGF-1-induced inflammation [84, 86]. Furthermore, dysregulated GH activates the MAPK signaling pathway and S6 kinase in 3T3-F442A preadipocytes [88]. Anderson et al. [88] proposed that GH may increase inflammatory reactions by activating the MAPK pathway.

**Fig. 4:**
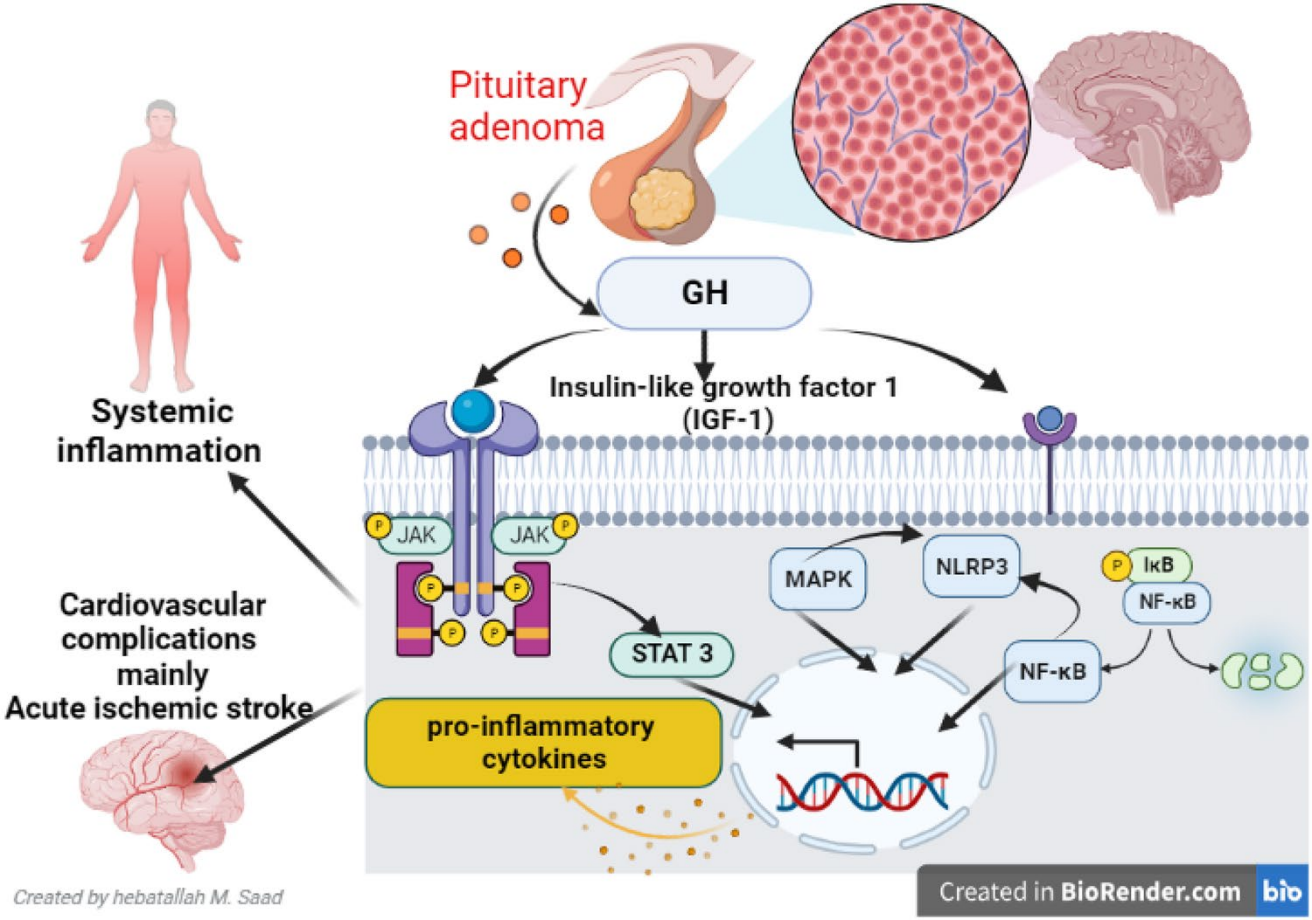
Acromegaly and inflammatory pathways: Growth hormone (GH) excess triggers the activation of signal transducer and activator of transcription 3 (STAT3), Janus kinase (JAK), nuclear factor kappa B (NF-κB), nod-like receptor pyrin 3 (NLRP3) and mitogen-activated protein kinase (MAPK) in acromegaly leading to systemic inflammation with the development of cardiovascular complications mainly acute ischemic stroke

Together with these findings, activated signaling pathways, including STAT3, NF-κB, NLRP3 inflammasome, and MAPK in acromegaly, may induce systemic inflammation with the development of cardiovascular complications, mainly AIS (Fig. 4).

## Conclusion

There is a potential link between acromegaly and the incidence of AIS due to acromegaly-induced hypertension, cardiometabolic disorders, inflammatory disorders, oxidative stress, and thrombotic complications. Local and systemic effects of pituitary adenoma affect brain integrity of brain circulation and systemic inflammatory complications, respectively. This review revealed that acromegaly triggers the development of AIS through local and systemic effects by inducing the formation of a cerebral vessel aneurysm, releasing pro-inflammatory cytokines, and developing oxidative stress, ED, and thrombosis correspondingly. However, this review cannot give a final sold conclusion concerning the link between acromegaly and the development of AIS. Herein, preclinical and clinical studies are warranted in this regard.

## Data Availability

All data are available in this manuscript.

## Abbreviations

GH: Growth hormone
IGF-I: Insulin-like growth factor I
AHR: Aryl hydrocarbon receptor
CT: Computed tomography
MRI: Magnetic resonance tomography imaging
OGTT: Oral glucose tolerance test
IHD: Ischemic heart disease
BBB: Blood–brain barrier
MMPs: Matrix metalloproteinase
IL-1β: Interleukins
TNF-α: Tumor necrosis factors
NF-κB: Nuclear factor kappa B
cAMP: Cyclic adenosine monophosphate
PDE1: Phosphodiesterase enzyme type 1
BDNF: Brain-derived neurotrophic factor
NFPA: Non-functional pituitary adenoma
SPECT: Single photon emission computed tomography
CSF: Cerebrospinal fluid
LDL: Low-density lipoprotein
VLDL: Very low-density lipoprotein
PAI-1: Plasminogen activator inhibitor 1
CRP: C reactive protein
TAFI: Thrombin-activatable fibrinolysis inhibitor,
NO: Nitric oxide
STAT3: Signal transducer and activator of transcription 3
JAK: Janus kinase
VCAM-1: Vascular cell adhesion molecules 1
NLRP3: Nod-like receptor pyrin 3
MAPK: Mitogen-activated protein kinase
IFNγ: Interferon Gamma

## References

[1] Melmed S, Bronstein MD, Chanson P, Klibanski A, Casanueva FF, Wass JA (2018). A consensus statement on acromegaly therapeutic outcomes. Nat Rev Endocrinol.

[2] Giustina A, Barkhoudarian G, Beckers A, Ben-Shlomo A, Biermasz N, Biller B (2020). Multidisciplinary management of acromegaly: a consensus. Rev Endocr Metab Disord.

[3] Lavrentaki A, Paluzzi A, Wass JA, Karavitaki N (2017). Epidemiology of acromegaly: review of population studies. Pituitary.

[4] Agustsson TT, Baldvinsdottir T, Jonasson JG, Olafsdottir E, Steinthorsdottir V, Sigurdsson G (2015). The epidemiology of pituitary adenomas in Iceland, 1955–2012: a nationwide population-based study. Eur J Endocrinol.

[5] Pearce J (2006). Nicolas Saucerotte: acromegaly before Pierre Marie. J Hist Neurosci.

[6] De Herder WW (2016). The history of acromegaly. Neuroendocrinology.

[7] Ribeiro-Oliveira A, Barkan A (2012). The changing face of acromegaly—advances in diagnosis and treatment. Nat Rev Endocrinol.

[8] Sabino SM, Miranda PAC, Ribeiro-Oliveira A (2010). Growth hormone-secreting pituitary adenomas: from molecular basis to treatment options in acromegaly. Cancer Biol Ther.

[9] Vilar L, Vilar CF, Lyra R, Lyra R, Naves LA (2017). Acromegaly: clinical features at diagnosis. Pituitary.

[10] Esposito D, Ragnarsson O, Johannsson G, Olsson DS (2020). Prolonged diagnostic delay in acromegaly is associated with increased morbidity and mortality. Eur J Endocrinol.

[11] AlMalki MH, Ahmad MM, Buhary BM, Aljawair R, Alyamani A, Alhozali A (2020). Clinical features and therapeutic outcomes of patients with acromegaly in Saudi Arabia: a retrospective analysis. Hormones.

[12] Gadelha MR, Kasuki L, Lim DS, Fleseriu M (2019). Systemic complications of acromegaly and the impact of the current treatment landscape: an update. Endocr Rev.

[13] Cannavo S, Ferrau F, Ragonese M, Romeo P, Torre M, Puglisi S (2014). Increased frequency of the rs2066853 variant of aryl hydrocarbon receptor gene in patients with acromegaly. Clin Endocrinol (Oxf).

[14] Sambugaro S, Di Ruvo M, Ambrosio MR, Pellegata NS, Bellio M, Guerra A (2015). Early onset acromegaly associated with a novel deletion in CDKN1B 5′ UTR region. Endocrine.

[15] Katznelson L, Laws ER, Melmed S, Molitch ME, Murad MH, Utz A (2014). Acromegaly: an endocrine society clinical practice guideline. J Clin Endocrinol Metab.

[16] Fleseriu M, Biller BM, Freda PU, Gadelha MR, Giustina A, Katznelson L (2021). A Pituitary Society update to acromegaly management guidelines. Pituitary.

[17] Holdaway I, Bolland M, Gamble G (2008). A meta-analysis of the effect of lowering serum levels of GH and IGF-I on mortality in acromegaly. Eur J Endocrinol.

[18] Hong S, Kim K-S, Han K, Park C-Y (2022). Acromegaly and cardiovascular outcomes: a cohort study. Eur Heart J.

[19] Colao A, Pivonello R, Grasso LFS, Auriemma RS, Galdiero M, Savastano S (2011). Determinants of cardiac disease in newly diagnosed patients with acromegaly: results of a 10 year survey study. Eur J Endocrinol.

[20] Dekkers O, Biermasz N, Pereira A, Romijn J, Vandenbroucke J (2008). Mortality in acromegaly: a metaanalysis. J Clin Endocrinol Metab.

[21] Al-Kuraishy HM, Al-Gareeb AI, Naji MT (2020). Brain natriuretic peptide in patients with acute ischemic stroke: Role of statins. Biomedical and Biotechnology Research Journal (BBRJ).

[22] Al-kuraishy H, Al-Gareeb A (2020) Vinpocetine and Ischemic Stroke. In: Pratap S (ed) Ischemic stroke. IntechOpen, Rijeka, p. Ch. 6

[23] Rücker V, Heuschmann PU, O’Flaherty M, Weingärtner M, Hess M, Sedlak C (2020). Twenty-year time trends in long-term case-fatality and recurrence rates after ischemic stroke stratified by etiology. Stroke.

[24] Al-Kuraishy HM, Al-Gareeb AI, Alblihed M, Cruz-Martins N, Batiha GE (2021). COVID-19 and risk of acute ischemic stroke and acute lung injury in patients with type II diabetes mellitus: the anti-inflammatory role of metformin. Front Med (Lausanne).

[25] Al-Thomali AW, Al-Kuraishy HM, Al-Gareeb AI, A KA-B, De Waard M, Sabatier JM et al (2022) Role of Neuropilin 1 in COVID-19 patients with acute ischemic stroke. Biomedicines 10(8):2032. 10.3390/biomedicines10082032 PMC9405641 36009579

[26] Al-Kuraishy HM, Al-Gareeb AI, Naji MT, Al-Mamorry F (2020). Role of vinpocetine in ischemic stroke and poststroke outcomes: a critical review. Brain Circulation.

[27] Al-Kuraishy HM, Al-Gareeb AI, Naji MT (2021). Statin therapy associated with decreased neuronal injury measured by serum S100β levels in patients with acute ischemic stroke. Int J Crit Illn Inj Sci Oct-Dec.

[28] Al-Kuraishy HM, Hussien NR, Al-Naimi MS, Al-Gareeb AI, Lugnier C (2021). Statins therapy improves acute ischemic stroke in patients with cardio-metabolic disorders measured by lipoprotein-associated phospholipase A2 (Lp-PLA2): new focal point. Neurol India.

[29] Naji MT, Al-Kuraishy HM, Al-Gareeb AI (2021). Differential effects of statins on matrix metalloproteinase-9 (MMP-9) in patients with acute ischaemic stroke: a potential for salutary. JPMA J Pak Med Assoc.

[30] Batiha GE-S, Gari A, Elshony N, Shaheen HM, Abubakar MB, Adeyemi SB (2021). Hypertension and its management in COVID-19 patients: the assorted view. Int J Cardiol Cardiovasc Risk Prev.

[31] Oh JS, Kim HJ, Hann HJ, Kang TU, Kim DS, Kang MJ (2021). Incidence, mortality, and cardiovascular diseases in pituitary adenoma in Korea: a nationwide population-based study. Pituitary.

[32] Mon SY, Alkabbani A, Hamrahian A, Thorton JN, Kennedy L, Weil R (2013). Risk of thromboembolic events in patients with prolactinomas compared with patients with non-functional pituitary adenomas. Pituitary.

[33] Klimenko LL, Skalny AV, Turna AA, Tinkov AA, Budanova MN, Baskakov IS (2016). Serum trace element profiles, prolactin, and cortisol in transient ischemic attack patients. Biol Trace Elem Res.

[34] Urban A, Masopust J, Maly R, Hosák L, Kalnická D (2007). Prolactin as a factor for increased platelet aggregation. Neuroendocrinol Lett.

[35] Edwards NJ, Kamel H, Josephson SA (2012). The safety of intravenous thrombolysis for ischemic stroke in patients with pre-existing cerebral aneurysms: a case series and review of the literature. Stroke.

[36] Pant B, Arita K, Kurisu K, Tominaga A, Eguchi K, Uozumi T (1997). Incidence of intracranial aneurysm associated with pituitary adenoma. Neurosurg Rev.

[37] Habibi Z, Miri SM, Sheikhrezaei A (2015). Pituitary macroadenoma coexistent with a posterior circulation aneurysm leading to subarachnoidal hemorrhage during transsphenoidal surgery. Turk Neurosurg.

[38] Satyarthee GD, Raheja A (2017). Unruptured internal carotid artery aneurysm associated with functional pituitary adenoma: a true association. Asian J Neurosurg Oct-Dec.

[39] Wakai S, Fukushima T, Furihata T, Sano K (1979). Association of cerebral aneurysm with pituitary adenoma. Surg Neurol.

[40] Jakubowski J, Kendall B (1978). Coincidental aneurysms with tumours of pituitary origin. J Neurol Neurosurg Psychiatr.

[41] Weir B (1992). Pituitary tumors and aneurysms: case report and review of the literature. Neurosurgery.

[42] Chen ML, Gupta A, Chatterjee A, Khazanova D, Dou E, Patel H (2018). Association between unruptured intracranial aneurysms and downstream stroke. Stroke.

[43] Li W, Zhu W, Wang A, Zhang G, Zhang Y, Wang K (2021). Effect of adjusted antiplatelet therapy on preventing ischemic events after stenting for intracranial aneurysms. Stroke.

[44] Spille DC, Vorona E, Catalino MP, Reuter G, Beckers A, Holling M (2023). Vascular anomalies in patients with growth hormone-secreting pituitary adenomas: illustrative case report and systematic review of the literature. Pituitary.

[45] Ngoepe MN, Pretorius E, Tshimanga IJ, Shaikh Z, Ventikos Y, Ho WH (2021). Thrombin-fibrinogen in vitro flow model of thrombus growth in cerebral aneurysms. TH Open.

[46] Ngoepe MN, Frangi AF, Byrne JV, Ventikos Y (2018). Thrombosis in cerebral aneurysms and the computational modeling thereof: a review. Front Physiol.

[47] Masuda H, Kondo K, Yokota K, Nemoto M, Kano T, Goto S (2008). Disturbed temporal lobe circulation due to compression by pituitary adenoma. Nippon Shimyureshon Geka Gakkai Kaishi.

[48] Plummer C, Henderson RD, O'Sullivan JD, Read SJ (2011). Ischemic stroke and transient ischemic attack after head and neck radiotherapy: a review. Stroke.

[49] Eseonu CI, ReFaey K, Geocadin RG, Quinones-Hinojosa A (2016). Postoperative cerebral vasospasm following transsphenoidal pituitary adenoma surgery. World Neurosurg.

[50] Ahn J-M, Oh H-J, Oh J-S, Yoon S-M (2020) Pituitary apoplexy causing acute ischemic stroke: which treatment should be given priority. Surg Neurol Int 11:113. 10.25259/SNI_82_2020 PMC7265385 32494388

[51] Sattler MG, Vroomen PC, Sluiter WJ, Schers HJ, van den Berg G, Langendijk JA (2013). Incidence, causative mechanisms, and anatomic localization of stroke in pituitary adenoma patients treated with postoperative radiation therapy versus surgery alone. Int J Radiat Oncol* Biol* Phys.

[52] Van Westrhenen A, Muskens I, Verhoeff J, Smith T, Broekman M (2017). Ischemic stroke after radiation therapy for pituitary adenomas: a systematic review. J Neurooncol.

[53] Brada M, Ashley S, Ford D, Traish D, Burchell L, Rajan B (2002). Cerebrovascular mortality in patients with pituitary adenoma. Clin Endocrinol (Oxf).

[54] Van Varsseveld N, Van Bunderen C, Ubachs D, Franken A, Koppeschaar H, van der Lely A-J (2015). Cerebrovascular events, secondary intracranial tumors, and mortality after radiotherapy for nonfunctioning pituitary adenomas: a subanalysis from the Dutch National registry of growth hormone treatment in adults. J Clin Endocrinol Metab.

[55] Murphy ES, Xie H, Merchant TE, Yu JS, Chao ST, Suh JH (2015). Review of cranial radiotherapy-induced vasculopathy. J Neurooncol.

[56] Reyes-Vidal CM, Mojahed H, Shen W, Jin Z, Arias-Mendoza F, Fernandez JC (2015). Adipose tissue redistribution and ectopic lipid deposition in active acromegaly and effects of surgical treatment. J Clin Endocrinol Metab.

[57] Vilar L, Naves LA, Costa SS, Abdalla LF, Coelho CE, Casulari LA (2007). Increase of classic and nonclassic cardiovascular risk factors in patients with acromegaly. Endocr Pract.

[58] de Almeida MCC, Freire CMV, Maria do Carmo PN, Soares BS, Barbosa MM, Giannetti AV (2022). Subclinical atherosclerosis in acromegaly: possible association with cardiovascular risk factors rather than disease activity. Growth Horm IGF Res.

[59] Erem C, Nuhoglu İ, Kocak M, Yilmaz M, Sipahi ST, Ucuncu O (2008). Blood coagulation and fibrinolysis in patients with acromegaly: increased plasminogen activator inhibitor-1 (PAI-1), decreased tissue factor pathway inhibitor (TFPI), and an inverse correlation between growth hormone and TFPI. Endocrine.

[60] Chuang S-Y, Bai C-H, Chen W-H, Lien L-M, Pan W-H (2009). Fibrinogen independently predicts the development of ischemic stroke in a Taiwanese population: CVDFACTS study. Stroke.

[61] Meng R, Li Z-Y, Ji X, Ding Y, Meng S, Wang X (2011). Antithrombin III associated with fibrinogen predicts the risk of cerebral ischemic stroke. Clin Neurol Neurosurg.

[62] Mendoza E, Malong CL, Tanchee-Ngo MJ, Mercado-Asis L (2015) Acromegaly with cardiomyopathy, cardiac thrombus and hemorrhagic cerebral infarct: a case report of therapeutic dilemma with review of literature. Int J Endocrinol Metab 13(2):e18841. 10.5812/ijem.18841 PMC4397949 25926851

[63] Elarabi AM, Mosleh E, Alamlih LI, Albakri MM, Ibrahim WH (2018). Massive pulmonary embolism as the initial presentation of acromegaly: is acromegaly a hypercoagulable condition?. American J Case Rep.

[64] Erdoğan M, Özbek M, Akbal E, Üreten K (2019). Plasma thrombin-activatable fibrinolysis inhibitor (TAFI) antigen levels in acromegaly patients in remission. Turkish J Med Sci.

[65] Colak A, Yılmaz H, Temel Y, Demirpence M, Simsek N, Karademirci I (2016). Coagulation parameters and platelet function analysis in patients with acromegaly. J Endocrinol Invest.

[66] Esposito D, Olsson DS, Franzén S, Miftaraj M, Nåtman J, Gudbjörnsdottir S (2022). Effect of diabetes on morbidity and mortality in patients with acromegaly. J Clin Endocrinol Metab.

[67] Anagnostis P, Efstathiadou Z, Gougoura S, Polyzos S, Karathanasi E, Dritsa P (2013). Oxidative stress and reduced antioxidative status, along with endothelial dysfunction in acromegaly. Horm Metab Res.

[68] Brevetti G, Marzullo P, Silvestro A, Pivonello R, Oliva G, Di Somma C (2002). Early vascular alterations in acromegaly. J Clin Endocrinol Metab.

[69] Rizzo M, Montalto G, Rizvi AA, Christ ER (2012). The role of elevated growth hormone on the increased atherosclerosis in patients with acromegaly.

[70] Wolters TLC, van der Heijden CDCC, van Leeuwen N, Hijmans-Kersten BTP, Netea MG, Smit J (2019). Persistent inflammation and endothelial dysfunction in patients with treated acromegaly. Endocr Connect.

[71] Kadhim SS, Al-Windy SA, Al-Kuraishy HM, Al-Gareeb AI (2019). Endothelin-1 is a surrogate biomarker link severe periodontitis and endothelial dysfunction in hypertensive patients: the potential nexus. J Int Oral Health.

[72] Chehaibi K, Trabelsi I, Mahdouani K, Slimane MN (2016). Correlation of oxidative stress parameters and inflammatory markers in ischemic stroke patients. J Stroke Cerebrovasc Dis.

[73] Tian R, Wu B, Fu C, Guo K (2020). miR-137 prevents inflammatory response, oxidative stress, neuronal injury and cognitive impairment via blockade of Src-mediated MAPK signaling pathway in ischemic stroke. Aging (Albany NY).

[74] Friedbichler K, Themanns M, Mueller KM, Schlederer M, Kornfeld JW, Terracciano LM (2012). Growth-hormone–induced signal transducer and activator of transcription 5 signaling causes gigantism, inflammation, and premature death but protects mice from aggressive liver cancer. Hepatology.

[75] Chhabra Y, Nelson CN, Plescher M, Barclay JL, Smith AG, Andrikopoulos S (2019). Loss of growth hormone–mediated signal transducer and activator of transcription 5 (STAT5) signaling in mice results in insulin sensitivity with obesity. FASEB J.

[76] Jayaraj RL, Azimullah S, Beiram R, Jalal FY, Rosenberg GA (2019). Neuroinflammation: friend and foe for ischemic stroke. J Neuroinflammation.

[77] Wolters T, van der Heijden C, Pinzariu O, Hijmans-Kersten B, Jacobs C, Kaffa C (2021). The association between treatment and systemic inflammation in acromegaly. Growth Horm IGF Res.

[78] Al-Shawk RS (2017). Evaluation of some pro-inflammatory and anti-inflammatory factors in patients with acromegaly. Mustansiriya Med J.

[79] Kim SE, Kim J, Lee J-Y, Lee S-B, Paik J-S, Yang S-W (2021). Octreotide inhibits secretion of IGF-1 from orbital fibroblasts in patients with thyroid-associated ophthalmopathy via inhibition of the NF-κB pathway. PLoS ONE.

[80] Doğan K, Yıldız ŞN, Sarıakçalı B, Duman G, Bolat S (2021). Elevated levels of Activin-A, TNF-Alpha and IL-6 in acromegaly. Neurochem J.

[81] Al-Kuraishy HM, Al-Gareeb AI, Saad HM, Batiha GE-S (2022) The effect of ramatroban on cytokine and thrombotic storms in Covid-19. Inflammopharmacology 2022/12/20 10.1007/s10787-022-01114-8 PMC9763788 36538271

[82] De Luca F (2020). Regulatory role of NF-κB in growth plate chondrogenesis and its functional interaction with Growth Hormone. Mol Cell Endocrinol.

[83] Liu Z, Yu Y, Jiang Y, Li J (2002). Growth hormone increases lung NF-κB activation and lung microvascular injury induced by lipopolysaccharide in rats. Ann Clin Lab Sci.

[84] Wolters TL, Netea MG, Riksen NP, Hermus AR, Netea-Maier RT (2020). Acromegaly, inflammation and cardiovascular disease: a review. Rev Endocr Metab Disord.

[85] Møller N, Jørgensen JOL (2009). Effects of growth hormone on glucose, lipid, and protein metabolism in human subjects. Endocr Rev.

[86] Maffei P, Dassie F, Wennberg A, Parolin M, Vettor R (2019). The endothelium in acromegaly. Front Endocrinol (Lausanne).

[87] Spadaro O, Goldberg EL, Camell CD, Youm Y-H, Kopchick JJ, Nguyen KY (2016). Growth hormone receptor deficiency protects against age-related NLRP3 inflammasome activation and immune senescence. Cell Rep.

[88] Anderson NG (1992). Growth hormone activates mitogen-activated protein kinase and S6 kinase and promotes intracellular tyrosine phosphorylation in 3T3-F442A preadipocytes. Biochem J.

